# New aspects characterizing non-obese NAFLD by the analysis of the intestinal flora and metabolites using a mouse model

**DOI:** 10.1128/msystems.01027-23

**Published:** 2024-02-29

**Authors:** Wenji Zhang, Wenli Cheng, JingHui Li, Zhenrui Huang, Hui Lin, Wenjuan Zhang

**Affiliations:** 1 Guangdong Provincial Engineering and Technology Research Center for Tobacco Breeding and Comprehensive Utilization, Key Laboratory of Crop Genetic Improvement of Guangdong Province, Crops Research Institute, Guangdong Academy of Agricultural Sciences, Guangzhou, China; 2 Department of Public Health and Preventive Medicine, School of Medicine, Jinan University, Guangzhou, China; 3 Department of Radiation Oncology, Guangdong Provincial People's Hospital (Guangdong Academy of Medical Sciences), Southern Medical University, Guangzhou, China; 4 Ningbo Psychiatric Hospital, Ningbo, China; University of Hawaii at Manoa, Honolulu, Hawaii, USA

**Keywords:** non-obese non-alcoholic fatty liver disease, intestinal flora, fecal metabolomics

## Abstract

Non-alcoholic fatty liver disease (NAFLD) is a major public health problem due to the high incidence affecting approximately one-third of the world’s population. NAFLD is usually linked to obesity and excessive weight. A subset of patients with NAFLD expresses normal or low body mass index; thus, the condition is called non-obese NAFLD or lean NAFLD. However, patients and healthcare professionals have little awareness and understanding of NAFLD in non-obese individuals. Furthermore, preclinical results from non-obese animal models with NAFLD are unclear. Gut microbiota and their metabolites in non-obese/lean-NAFLD patients differ from those in obese NAFLD patients. Therefore, we analyzed the biochemical indices, intestinal flora, and intestinal metabolites in a non-obese NAFLD mouse model established using a methionine-choline-deficient (MCD) diet. The significantly lean MCD mice had a remarkable fatty liver with lower serum triglyceride and free fatty acid levels, as well as higher alanine transaminase and aspartate transaminase levels than normal mice. 16S RNA sequencing of fecal DNA showed that the overall richness and diversity of the intestinal flora decreased in MCD mice, whereas the Firmicutes:Bacteroidota ratio was increased. g_*Tuzzerella*, s_*Bifidobacterium pseudolongum*, and s_*Faecalibaculum rodentium* were the predominant species in non-obese NAFLD mice. Fecal metabolomics using liquid chromatography-tandem mass spectrometry revealed the potential biomarkers for the prognosis and diagnosis of non-obese NAFLD, including high levels of tyramine glucuronide, 9,12,13-TriHOME, and pantetheine 4′-phosphate, and low levels of 3-carbamoyl-2-phenylpropionaldehyde, N-succinyl-L,L-2,6-diaminopimelate, 4-methyl-5-thiazoleethanol, homogentisic acid, and estriol. Our findings could be useful to identify and develop drugs to treat non-obese NAFLD and lean NAFLD.

**IMPORTANCE:**

Patients and healthcare professionals have little awareness and understanding of NAFLD in non-obese individuals. In fact, about 40% of people with NAFLD worldwide are non-obese, and nearly one-fifth are lean. Lean NAFLD unfortunately may be unnoticed for years and remains undetected until hepatic damage is advanced and the prognosis is compromised. This study focused on the lean NAFLD, screened therapeutic agents, and biomarkers for the prognosis and diagnosis using MCD-induced male C57BL/6J mice. The metabolites tyramine glucuronide, 9,12,13-TriHOME, and pantetheine 4′-phosphate, together with the predominant flora including g_*Tuzzerella*, s_*Bifidobacterium pseudolongum*, and s_*Faecalibaculum rodentium*, were specific in non-obese NAFLD mice and might be used as targets for non-obese NAFLD drug exploration. This study is particularly significant for non-obese NAFLDs that need to be more actively noticed and vigilant.

## INTRODUCTION

Non-alcoholic fatty liver disease (NAFLD) is the most common chronic liver disease characterized by an excessive deposition of fat in liver cells caused by pathogenic factors, except alcohol, which lead to liver damage. Chronic excessive accumulation of fat in the liver leads to NAFLD accompanied by non-alcoholic steatohepatitis (NASH) and liver fibrosis, further progressing into cirrhosis or liver cancer (1). It is also associated with type 2 diabetes mellitus (T2DM), cardiovascular disease, and other metabolic disorders. Individuals with NAFLD are two to five times more likely to develop T2DM than those without NAFLD (2), and cardiovascular disease is the most common cause of death in patients with NAFLD (3). According to the latest statistics, the incidence of NAFLD worldwide was 32.4% in May 2021 (4). This incidence in China was 29.2%, which was higher and more alarming than that in Europe and the United States (5). Approximately 40% of people with NAFLD worldwide are non-obese, and nearly one-fifth are lean (6). Moreover, despite the absence of obesity, several studies indicate that individuals with lean NAFLD have an increased risk of developing T2DM and all-cause mortality than those with obese NAFLD (7). Recent studies mostly focus on obese subjects, but the population, the patients themselves, and healthcare professionals have poor awareness and understanding of lean NAFLD. Lean NAFLD, unfortunately, may be unnoticed for years and can remain undetected until hepatic damage is advanced and the prognosis is compromised. At present, no drug has been approved by the Food and Drug Administration to treat NAFLD. Although several drugs have been developed to treat NAFLD in phase III trials, none have been studied in lean-NAFLD patients. Moreover, the results of drug development for lean NAFLD and application in animal models have also not been fully understood.

Animal models for lean NAFLD should account not only for weight and insulin resistance but also for pathological patterns and histological changes. Dietary induction models, including those established using a methionine-choline-deficient (MCD) diet, choline-deficient–amino acid-defined diet, and high-fat–high-fructose diet, as well as the combinations of such diets, have been most extensively used in previous studies (8). The MCD diet causes histological changes similar to those observed in human steatohepatitis, and it can develop into fibrosis (9). Usually, MCD-fed mice develop steatohepatitis after 3 weeks and liver fibrosis after 5 weeks. The body weight decreases by about 40% after 8 weeks (8, 9). Although the pathogenesis of this model is relatively well studied, the changes in their intestinal microenvironment including intestinal flora and metabolites are not clear enough.

Multiple factors and molecular pathways are involved in the development of NAFLD, including visceral obesity, lipodystrophy-like phenotype, diabetes mellitus, insulin resistance, *de novo* adipose formation, intestinal disorders, genetic factors, and epigenetic modifications (10, 11). The effects of these multiple pathogenic factors on a genetically susceptible subject are parallel or sequential, or in some way synergistic (12). The role of the gut microbiota has been increasingly implicated in the modulation of the risk factors of NAFLD, such as energy homeostasis dysregulation, insulin resistance, increase in intestinal permeability, endogenous production of ethanol, inflammation, as well as choline and bile acid metabolism (13). It is worth noting that lean and non-obese NAFLDs have some unique characteristics underlying intestinal microbial disorders and metabolite reconstruction (7). A demographic and clinical study of lean-NAFLD subjects (14) showed a significantly different microbial community in their fecal microbiota, and the decrease in Desulfovibrionaceae was associated with NAFLD in the lean-NAFLD group but not in the obese NAFLD group. Additionally, a pilot study on patients who underwent biopsy revealed that lean NASH individuals had a lower abundance of *Faecalibacterium*, *Ruminococcus*, and *Lactobacillus* than non-lean individuals with NASH (15). Another study conducted in a Chinese population demonstrated that non-obese patients with NAFLD exhibited a reduction in Firmicutes, including Lachnospiraceae, Ruminococcaceae, and Lactobacillaceae, and an increase in lipopolysaccharide-producing Gram-negative bacteria (16).

Therefore, in this study, an animal model of lean NAFLD was induced using an MCD diet, and the changes in serum and liver biochemical indices were analyzed. Moreover, the intestinal microenvironment, including the intestinal flora and its metabolites, was also studied to provide a reference for the diagnosis and treatment of NAFLD and for the development of appropriate drugs.

## MATERIALS AND METHODS

### Mice and treatment

Seven-week-old specific pathogen-free C57BL/6J (Guangzhou, SCXK [Yue] 2022-0002) male mice were purchased from the Guangdong Medical Laboratory Animal Center (Foshan, China). The mice were subjected to a 12 h/12 h light/dark cycle and a constant temperature (22°C ± 2°C) and were provided free access to a standard chow diet and water. After 7 days of adaptive feeding, the mice were randomly divided into a normal control (NC) group fed a standard diet (CAS: 1010009; Jiangsu Xietong Pharmaceutical Bio-engineering Co., Ltd.) (*n* = 6) and NAFLD mice fed the MCD diet (CAS: MCD; Shanghai Yitong Biotechnology Co., Ltd.) (*n* = 6) for 60 days. The body weights of mice were recorded during feeding every 3 days. The stools of mice were collected before sacrifice. All mice were fasted overnight at the end of the prescribed feeding period. The whole blood was collected from the retro-orbital plexus, allowed to stand at room temperature for at least 30 min, and then centrifuged (2,500 rpm for 20 min) to obtain the serum. A small portion of the freshly isolated and weighed livers was fixed in 4% paraformaldehyde, and the remaining liver samples and sera were immediately frozen in liquid nitrogen and stored at −80°C until further analysis.

### Biochemical and histopathological analyses

The biochemical indicators in the serum and liver were measured using the following kits: triglyceride (TG) (A110-1-1), total cholesterol (TC) (A111-1-1), high-density lipoprotein cholesterol (HDL-c) (A112-1-1), low-density lipoprotein cholesterol (A113-1-1), free fatty acid (FFA) (A042-2-1), alanine aminotransferase (ALT) (C009-2-1), and aspartate aminotransferase (AST) (C010-2-1) (all from Nanjing Jiancheng Bioengineering Institute, Nanjing, China).

The fixed livers were dehydrated using graded concentrations of alcohol, embedded in paraffin, and cut into 4-μm-thick slides, deparaffinized, rehydrated, and stained with hematoxylin and eosin using a standard protocol (cat # CO105S; Beyotime Biotechnology, Shanghai, China). The slides were observed using a motorized multifunctional upright fluorescence microscope (DM6000B; Leica, Germany).

### Determination of biomarkers of inflammation using enzyme-linked immunosorbent assay

Mouse enzyme-linked immunosorbent assay kits for the determination of tumor necrosis factor α (TNF-α) (MM-0132M1), interleukin-β (IL-1β) (MM-0040M1), IL-6 (MM-0163M1), and IL-10 (MM-0176M1) were purchased from Jiangsu MeiMian Industrial Co., Ltd (Yancheng, China). Briefly, the serum and standard were separately added to a 96-well plate. Then, horseradish peroxidase-conjugated reagent was added to each well and incubated for 60 min at 37°C. After washing five times with the wash buffer, chromogen solutions were added to each well, mixed gently, and incubated for 15 min at 37°C away from light. Lastly, the stop buffer was added to the 96-well plate, and the absorbance of each well was measured using an Imark microplate reader.

### 16S rRNA sequencing and bioinformatics analysis

#### Sample preparation

Total DNA was extracted (Omega Bio-Tek, Norcross, GA, USA) from 50- to 100-mg fecal samples and amplified using PCR with the primers 338F: ACTCCTACGGGAGGCAGCAG and 806R: GGACTACNNGGGTATCTAAT targeting the 16S V3–V4 rRNA gene with indexing barcodes. AxyPrep DNA Gel Extraction kit (Axygen Biosciences, Union City, CA, USA) was used to purify the recovered products, and the proteins were separated using 2% agarose gel electrophoresis. Proteins were detected and quantified using Qubit (v.4.0; Thermo Fisher Scientific, USA). The NEXTFLEX Rapid DNA-Seq Kit was used to build the library. Purified amplicons were pooled in equimolar amounts and paired-end sequenced on the Illumina PE300 platform (Illumina, San Diego, USA) according to the standard protocols of Majorbio Bio-Pharm Technology Co. Ltd. (Shanghai, China).

#### Sequence analysis

The 16S rRNA sequencing data have been made publicly available on the National Center for Biotechnology Information database (accession number PRJNA1064694). In our study, raw sequence tags were generated from FLASH (v.1.2.11, https://github.com/OpenGene/fastp). Quality filtering on the basic tags was performed under specific conditions to obtain high-quality clean labels according to the QIIME quality-controlled process (v.1.9.1, http://qiime.org/install/index.html). The tags were compared with the reference database (Gold database) using the UCHIME algorithm (UCHIME, 2019) to detect the chimera sequences that were removed, and effective tags were finally obtained. *De novo* operational taxonomic unit (OTU) clustering was carried out using Uparse (v.11, http://www.drive5.com/uparse/), which identifies highly accurate OTUs from amplicon sequencing data with an identity threshold of 97%. The taxonomy of each OTU representative sequence was analyzed using RDP Classifier (v.2.13, https://sourceforge.net/projects/rdp-classifier/) against the 16S rRNA gene database using a confidence threshold of 0.7. OTUs with an alignment ratio lower than 0.8 were excluded. Usearch (v.11, http://www.drive5.com/usearch/) was used for OTU count. Next, the OTUs were used to find the effective sequences using Mothur (https://www.mothur.org/wiki/Download_mothur).

The representative sequences of OTUs were used to analyze the alpha-diversity (Chao1, ACE, Coverage, Sobs, Shannon, and Simpson diversity indices) based on the relative abundances. The similarities among microbial communities in different samples were determined using principal coordinate analysis (PCoA) based on Bray-Curtis dissimilarity using the Vegan (v.2.5–3) package. The permutational multivariate analysis of variance test in the Vegan package was used to assess the percentage of variation due to the treatment, along with its statistical significance. Linear discriminant analysis effect size (LEfSe) was performed to identify the significantly abundant taxa (phylum to genera) of bacteria among different groups (linear discriminant analysis [LDA] score >2, *P* < 0.05). The variance inflation factor for each variable was estimated using the vif function in the car package (https://cran.r-project.org/web/packages/car/car.pdf).

### Ultra-high-performance liquid chromatography–tandem mass spectrometry of fecal metabolites

#### Sample preparation

Feces (100 mg) were added to a 2-mL centrifuge tube, and a 6-mm-diameter grinding bead was added. Extraction solution (400 µL, methanol:water = 4:1 [vol/vol]) containing 0.02 mg/mL of the internal standard (L-2-chlorophenylalanine) was used for metabolite extraction. Samples were ground using a Wonbio-96c frozen tissue grinder (Shanghai Wanbo Biotechnology Co., Ltd.) for 6 min (–10°C, 50 Hz), followed by low-temperature ultrasonic extraction for 30 min (5°C, 40 kHz). The samples were left at –20°C for 30 min and centrifuged for 15 min (4°C; 13,000 × *g*), and the supernatant was transferred to an injection vial for liquid chromatography–tandem mass spectrometry (LC-MS/MS). A pooled quality control (QC) sample was prepared by mixing equal volumes of all samples and injecting at regular intervals (every 5–15 samples) for system conditioning and QC.

#### Chromatographic conditions

LC-MS/MS was performed using a Thermo UHPLC-Q Exactive HF-X system equipped with an ACQUITY HSS T3 column (100 mm × 2.1 mm internal diameter, 1.8 µm; Waters Corporation, Milford, MA, USA) at Majorbio Bio-Pharm Technology Co. Ltd. The mobile phases consisted of 0.1% formic acid in water:acetonitrile (95:5, vol/vol) (solvent A) and 0.1% formic acid in acetonitrile:isopropanol:water (47.5:47.5, vol/vol) (solvent B). The other parameters were as follows: flow rate, 0.40 mL/min; column temperature, 40℃; and injection volume, 1 µL in both positive and negative ion modes. The mobile phase gradient for the positive ion mode was as follows: 0–3 min at 0%–20% B; 3.0–4.5 min at 10%–35% B; 4.5–5.0 min at 35%–100% B; 5.0–6.3 min at 100% B; 6.3–6.4 min at 100%–0% B; and 6.4–8.0 min at 0% B. The separation gradient in the negative ion mode was as follows: 0–1.5 min at 0%–5% B; 1.5–2.0 min at 5%–10% B; 2.0–4.5 min at 10%–30% B; 4.5–5.0 min at 30%–100% B; 5.0–6.3 min at 100% B; 6.3–6.4 min at 100%–0% B; and 6.4–8.0 min at 0% B.

#### Mass spectrometry conditions

Mass spectrometry data were collected using a Thermo UHPLC-Q Exactive HF-X mass spectrometer equipped with an electrospray ionization source operating in both positive and negative modes. The optimal conditions were as follows: source temperature, 425℃; sheath gas flow rate, 50 arb; aux gas flow rate, 13 arb; ion-spray voltage floating, –3,500 V in the negative mode and 3500 V in the positive mode; normalized collision energy, 20–40–60 V rolling for tandem mass spectrometry (MS/MS); full mass spectrometry resolution: 60,000; and MS/MS resolution: 7,500. Data acquisition was performed in the data-dependent acquisition mode. The detection was performed over a mass range of 70–1,050 *m*/*z*.

#### Substance identification and analysis

The liquid chromatography–mass spectrometry (LC/MS) raw data from our study have been made available on the MetaboLights database, under the accession number MTBLS9365. In our study, pretreatment of LC/MS raw data was performed using Progenesis QI software (Waters Corporation) through baseline filtering, peak identification, integration, retention time correction, and peak alignment. Internal standard peaks, as well as any known false-positive peaks (including noise, column bleed, and derivatized reagent peaks), were removed from the data matrix as deredundant and peak pooled. The metabolites were identified using the Human Metabolome Database (HMDB) (http://www.hmdb.ca/) and the Metlin (https://metlin.scripps.edu/) database.

At least 80% of metabolic features detected in any set of samples in the data matrix were retained. The minimum metabolite value was estimated after filtering specific samples with metabolite levels below the lower limit of quantification, and each metabolic signature was normalized to the sum. The response intensity of mass spectrometry peaks of the sample was normalized using the sum-normalization method to reduce errors caused by sample preparation and instrument instability. Furthermore, variables of QC samples with relative standard deviation of >30% were excluded and subjected to log_10_ transformation to obtain the final data matrix for subsequent analysis.

Next, principal component analysis using the R package “ropls” (v.1.6.2), orthogonal partial least squares discriminant analysis (OPLS-DA), and seven-cycle interactive validation was performed to evaluate model stability. Metabolites with variable importance in projection (VIP) of >1, *P* < 0.05, and one-time fold change were determined as significantly different metabolites based on the VIP values obtained using the OPLS-DA model, and the *P* value was generated using Student’s *t*-test. The Kyoto Encyclopedia of Genes and Genomes (KEGG) database was used for metabolic pathway annotation. Python package “scipy.stats” (https://docs.scipy.org/doc/scipy/) was used to perform enrichment analysis to obtain the most relevant biological pathways for experimental treatments using Fisher exact test.

### Statistical analysis

Statistical analysis was performed using GraphPad Prism (v.7.0) for Windows (GraphPad Software Inc., San Diego, CA, USA). Groups were compared using Student’s *t*-test. Results are presented as mean ± standard deviation of at least three independent experiments. A *P* value of <0.05 was considered statistically significant.

## RESULTS

### Successful establishment of the lean-NAFLD model

**Fig 1:**
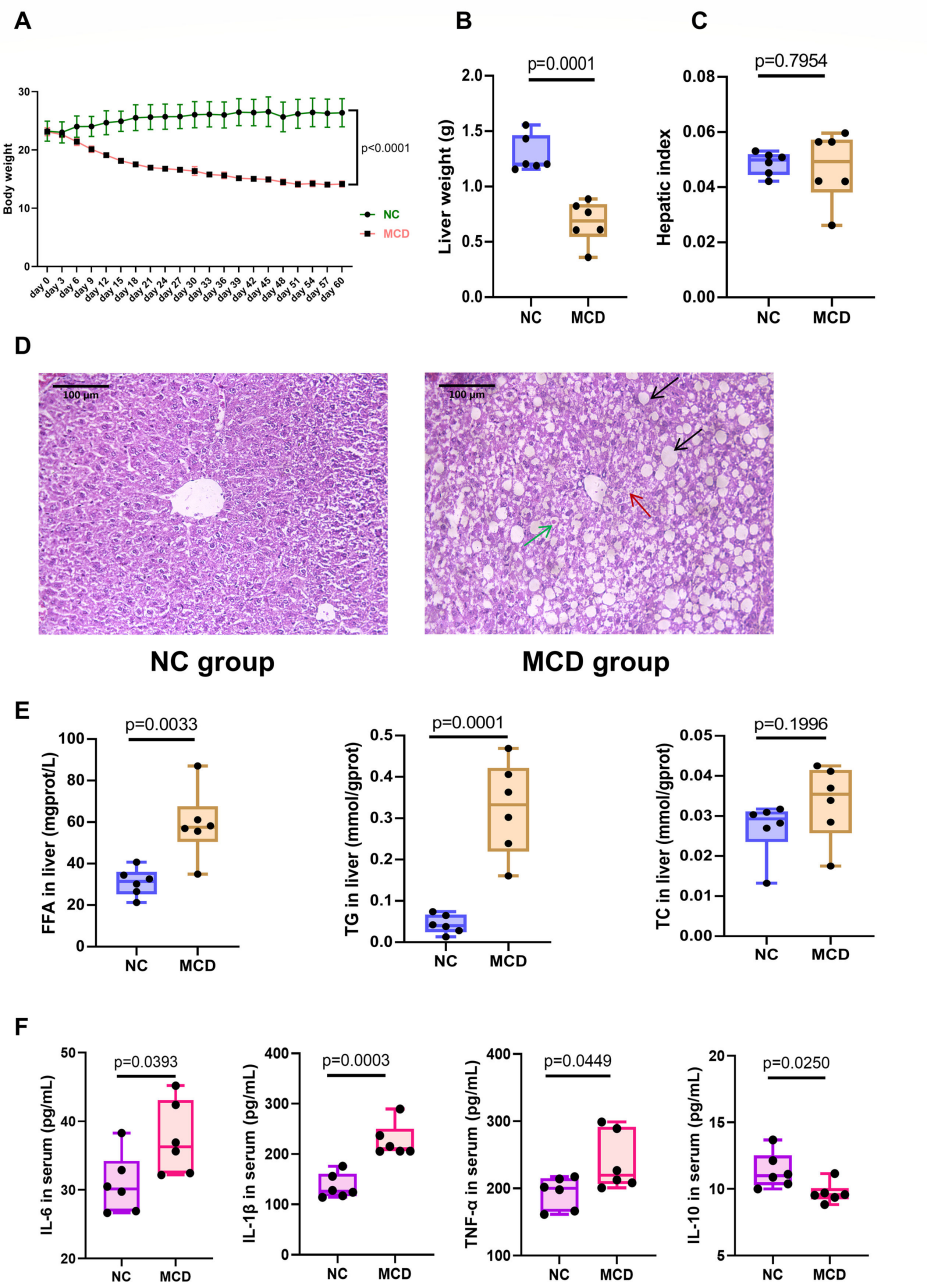
Body and liver weight, liver histopathology, lipid content, and inflammation state of mice in the normal control (NC) and NAFLD (MCD) groups. (**A**) Body weight changes in NC and MCD mice after 60 days. (**B and C**) Liver weight and hepatic index. (**D**) Representative pictures of hematoxylin and eosin staining of the liver (×400 magnification). Dark arrows indicate hepatic cell steatosis; green arrow indicates hepatic cell acidophilic necrosis; and red arrow indicates focal necrosis. (**E**) Changes in lipid levels (free fatty acid [FFA], triglycerides [TGs], and total cholesterol [TC]) in the liver of control mice and mice with NAFLD. (**F**) Levels of inflammatory factors (TNF-α, IL-1β, IL-6, and IL-10) in serum.

**Fig 2:**
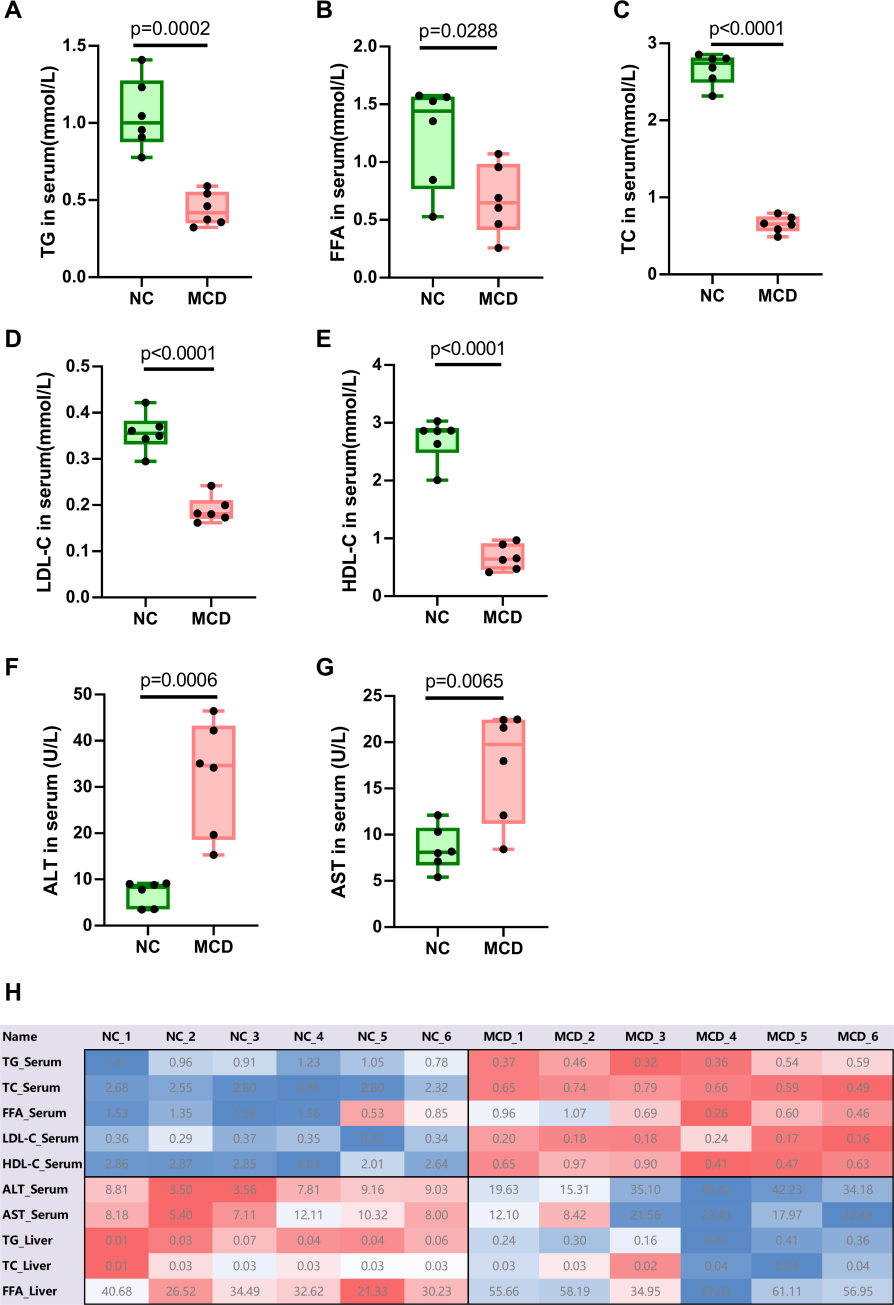
Serum lipid profiles and liver function indicators in normal control (NC) and NAFLD (MCD) mice. (**A**) Triglyceride (TG), (**B**) total cholesterol (TC), (**C**) free fatty acid (FFA), (**D**) low-density lipoprotein cholesterol (LDL-c), (**E**) high-density lipoprotein cholesterol (HDL-c) (**F**) alanine aminotransferase (ALT), and (**G**) aspartate aminotransferase (AST). (**H**) Change profile of lipid index and liver function enzyme index, where the numbers indicate the measured averages.

The body weight of NC mice at the end of the experiment did not change significantly (26.38 ±  2.40 g), whereas that of MCD-fed mice decreased significantly (14.27 ± 0.56 g) (*P* < 0.0001) (Fig. 1A). MCD feeding also caused a significant decrease in liver weight (*P* = 0.0001) (Fig. 1B), which was proportionally associated with weight loss, as the liver weight-to-body weight ratio in MCD-fed mice was comparable with that of mice fed a normal diet (Fig. 1C). Hepatic cell vacuolation and steatosis were observed in the livers of mice in the MCD group, which was consistent with the fatty liver phenotype (Fig. 1D). Some hepatic cells showed evident ballooning and swelling, with inflammatory infiltration in the hepatic lobes. Hepatic cell necrosis was also observed around the cracks. The liver cells of mice in the NC group were polygonal and arranged as hepatic cords and radially distributed around the central vein. The centers of cells were occupied by large and round nuclei; the cells had uniform cytoplasm, no lipid droplets, and no steatosis or inflammatory cell infiltration. An increase in TG and FFA levels in the livers of mice in the MCD group was observed (*P* < 0.01), whereas no significant changes were noted in TC levels (Fig. 1E). The levels of the proinflammatory factors TNF-α, IL-6, and IL-1β were obviously upregulated in MCD-fed mice compared with those in the NC group (*P* < 0.05), whereas the anti-inflammatory cytokine IL-10 decreased significantly in MCD-fed mice (*P* = 0.025) (Fig. 1F). The levels of TC, TG, FFA, LDL, and high-density lipoprotein (HDL) in the serum were lower in the MCD group than in the NC group (Fig. 2A through E). The liver function enzymes ALT and AST (Fig. 2F and G) were significantly increased, indicating that fat accumulation and degeneration seriously affected the normal function of the liver. These results indicated that the MCD diet had successfully induced a lean mouse model of NAFLD.

### High-throughput 16S rRNA sequencing

**Fig 3:**
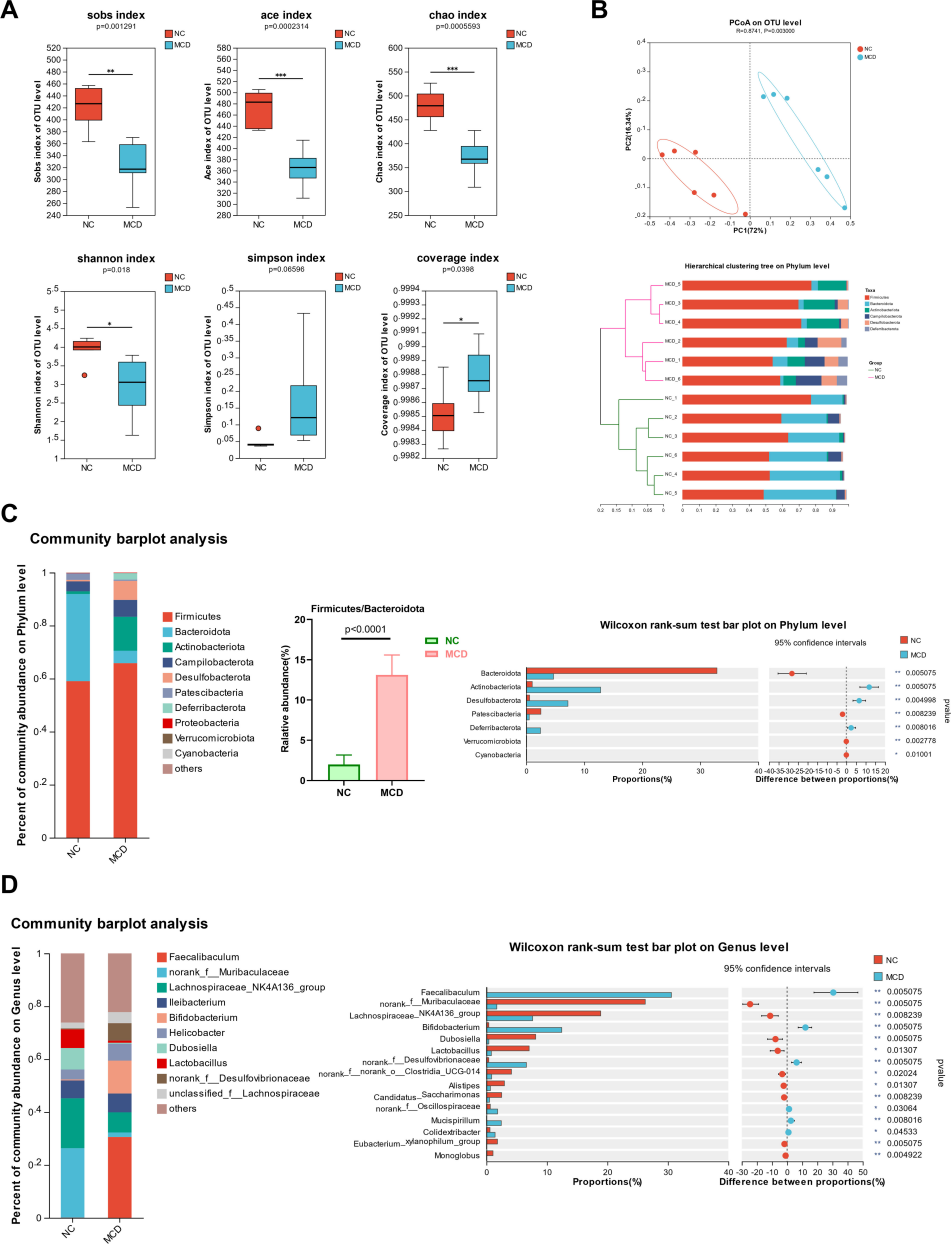
Changes in gut microbiota composition. (**A**) Alpha-diversity analysis including Sobs, ACE, Chao, Shannon, Simpson, and coverage indices of the bacterial community in the feces of normal control (NC) mice and mice with NAFLD (MCD). (**B**) Beta-diversity analysis between NC mice and MCD using principal coordinate analysis (PCoA) and hierarchical clustering analysis. (**C**) Relative abundance of bacteria at the phylum level, ratio of Firmicutes to Bacteroidota, and Wilcoxon rank-sum test to compare differences between the NC and MCD groups at the phylum level. (**D**) Relative abundance of bacteria at the genus level, and the Wilcoxon rank-sum test to compare differences between the NC and MCD groups at the genus level.The symbols *, **, and *** indicate that the *P*-value is less than 0.05, 0.01, and 0.001, respectively.

The alpha-diversity of intestinal microbiota was evaluated using Chao, ACE, Shannon, coverage, Sobs, and Simpson indices in normal mice and those with NAFLD. Sobs index reflects the actual observed richness, ACE and Chao indices reflect the community richness, Shannon and Simpson indices reflect the community diversity, and the Good’s Coverage index estimates the proportion of individuals in the (whole) community that belong to the species present in a sample. Figure 3A shows that the Sobs, ACE, Chao, and Shannon indices of MCD-fed mice were significantly lower than those of NC mice. The Simpson index of lean-NAFLD mice did not change significantly. The coverage index was significantly higher in MCD-fed mice compared with that in NC mice. These results indicated that the diversity and abundance of microbial species in the feces of MCD-induced lean mouse model of NAFLD were reduced. PCoA and hierarchical clustering based on weighted UniFrac distances were performed to reveal differences in the bacterial community structure of samples (Fig. 3B). PCoA of OTU levels using the ANOSIM test (R = 0.8741, *P* = 0.003) showed that the gut microbiome composition among the NC and MCD groups was separated. In agreement with the findings from PCoA, clustering analysis showed that the NC and MCD groups showed clustering in their own groups. The composition of the intestinal microbiota at the phylum and genus level was analyzed to reveal the relative abundance of the main bacteria. The top 10 phyla and genera with the highest abundance are shown in Fig. 3C and D. The dominant phyla were Firmicutes, Bacteroidota, Actinobacteriota, Campilobacterota, and Desulfobacterota. The relative abundance of Bacteroidota and Patescibacteria was significantly higher in NC mice than in MCD-fed mice. The relative abundance of Actinobacteriota, Desulfobacterota, and Deferribacterota was significantly higher in MCD-fed mice than in NC mice. The ratio of Firmicutes to Bacteroidota was higher in MCD-fed mice (13.1 ± 2.47) than in NC mice (1.99 ± 1.19). The relative abundance of the dominant 10 genera was 78%–74%. Six genera, including *Faecalibaculum*, *Bifidobacterium*, *norank_f__Desulfovibrionaceae*, *norank_f__Oscillospiraceae*, *Mucispirillum*, and *Colidextribacter*, were significantly increased in the MCD group than in the NC group. The relative abundance of seven genera, including *norank_f__Muribaculaceae*, *Lachnospiraceae_NK4A136_group*, *Dubosiella*, *Lactobacillus*, *norank_f__norank_o__Clostridia_UCG-014*, *Alistipes*, and *Candidatus_Saccharimonas*, was significantly reduced in the MCD group.

**Fig 4:**
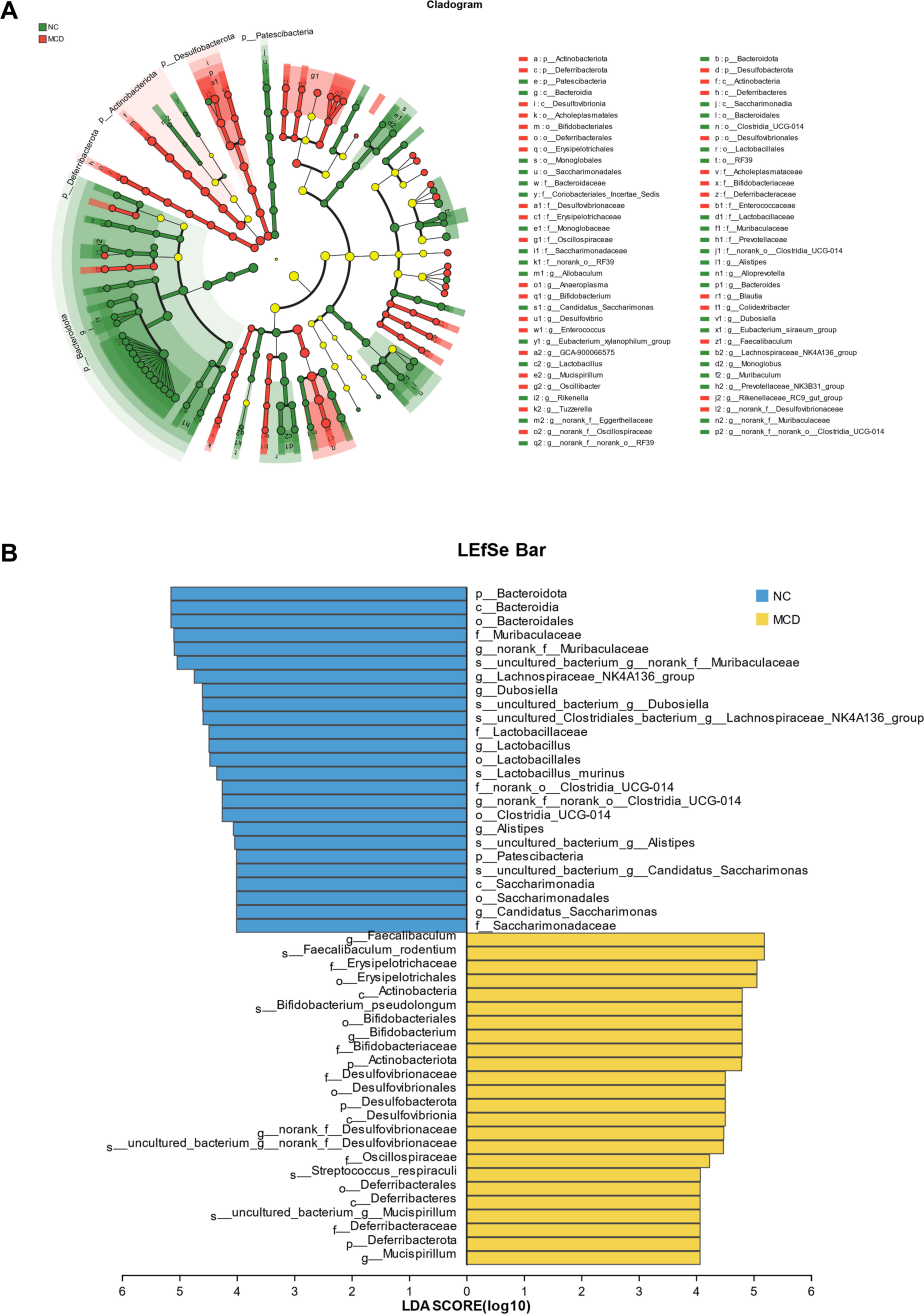
Linear discriminant analysis effect size (LEfSe) of microbiota. (**A**) LEfSe multilevel species at the level tree between the normal control (NC) and NAFLD (MCD) groups (*α* = 0.05, logarithmic linear discriminant analysis [LDA] score threshold = 3.0). Different color nodes representing the microbial groups significantly enriched in the corresponding group and with a significant effect on the difference between groups. Light yellow nodes represent the microbial groups that do not significantly differ between the two groups or have no significant effect on the differences between the two groups. if the number of significantly different species is <50, the legend position is displayed as one column on the right side; if the number of significantly different species is >50, the legend position is displayed as two columns on the right side. (**B**) LDA discrimination results between the NC and MCD groups (*α* = 0.05, LDA score threshold = 4.0). LDA discriminant histogram statistics highlighting the microbial groups with significant effects in different NC and MCD groups. The LDA score obtained using LDA indicates that the greater the LDA score, the greater the influence of species abundance on the difference effect.

LEfSe results (Fig. 4) showed that species with LDA scores of >4 were considered biological markers. The NC and MCD groups did not have common biomarkers at the noted genus levels. The biomarkers in NC mice at the noted genus level were *Lachnospiraceae_NK4A136_group*, *Dubosiella*, *Lactobacillus*, *Alistipes*, and *Candidatus_Saccharimonas*. The biomarkers of MCD-fed mice at the noted genus level were *Faecalibaculum*, *Bifidobacterium*, and *Mucispirillum*. The noted species in the NC group were *Lactobacillus murinus*, whereas *Streptococcus respiraculi* and *Bifidobacterium pseudolongum* were those in the MCD group. These results implied that abnormal intestinal flora played a key role in the development of NAFLD and represented an important factor leading to NAFLD.

### Fecal metabolomics profiling of MCD-induced NAFLD in mice

**Fig 5:**
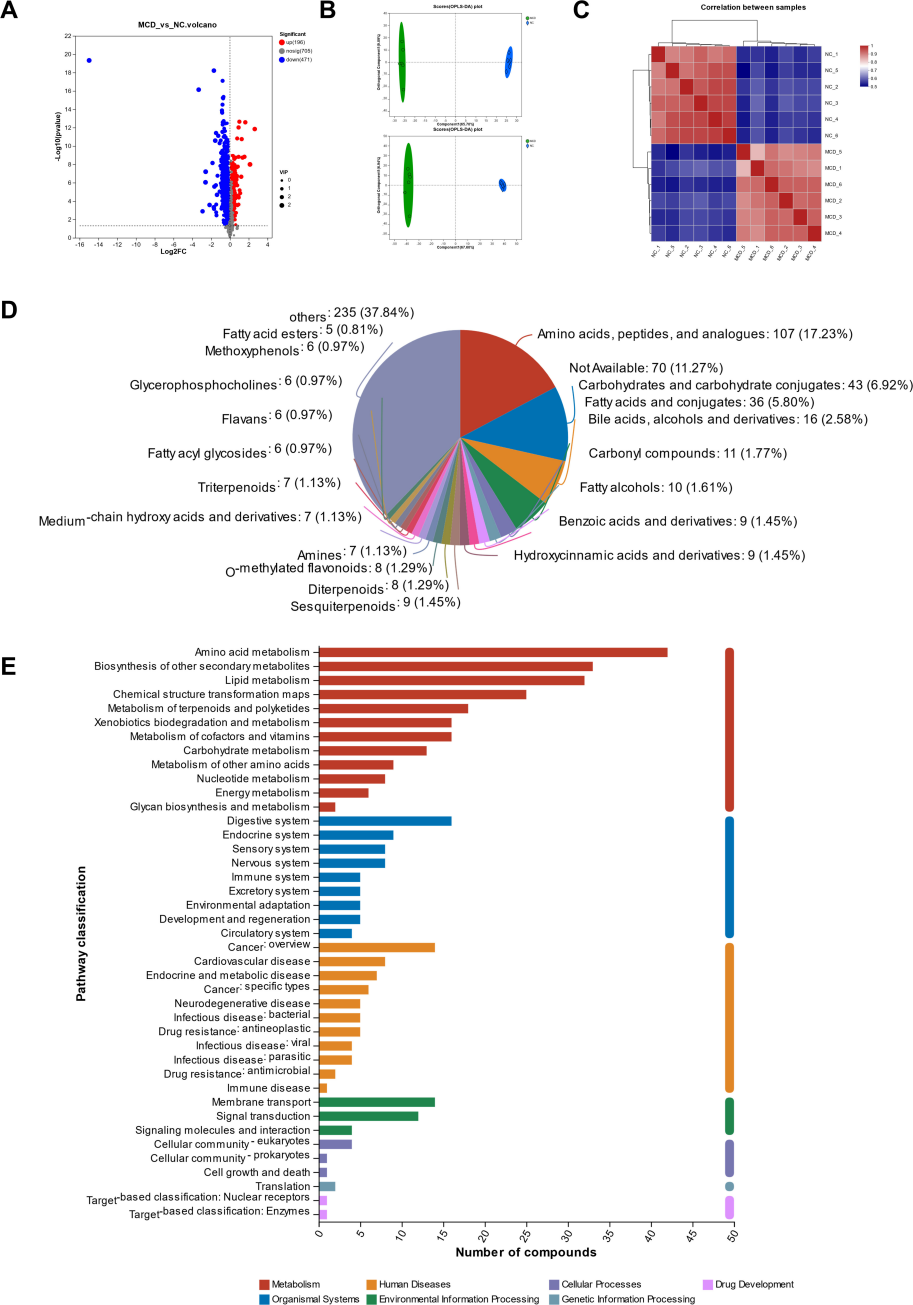
Metabolic profiles of intestinal flora. (**A**) Volcano plots of the differential metabolites between the normal control (NC) and NAFLD (MCD) groups in both positive and negative ion modes. The horizontal coordinate log2FC is the multiple change value of the difference in metabolite expression between the two groups; the vertical coordinate −log_10_ (*P* value) is the statistical test value of the difference in metabolite expression; the higher the value, the more significant the difference in expression. Each dot in the diagram represents a specific metabolite, and the size of the dot represents the variable importance in projection value. (**B**) OPLS-DA score plots of metabolites in each experimental group in the positive ion mode (upper) and negative ion mode (lower). (**C**) Correlations between samples using Pearson’s correlation coefficient by Euclidean distance algorithm. (**D**) Classification of the identified metabolites using the Human Metabolome Database. (**E**) Kyoto Encyclopedia of Genes and Genomes functional prediction analysis of the identified intestinal metabolites.

A total of 1,372 metabolites were identified in the feces in both positive and negative ion modes (Fig. 5A). A total of 667 metabolites were significantly different (*P* < 0.05, VIP_pred_OPLS-DA >1 and up/down fold change >1): 471 were upregulated in the NC group and 196 were upregulated in the MCD group. These metabolites were clustered using OPLS-DA, and their validation plots were obtained using 200 permutation tests. The variation degrees of metabolite composition and abundance between the NC and MCD groups were quantitatively analyzed using correlation data. The closer the correlation is to 1, the higher the similarity of metabolic composition and abundance between samples. The results showed that the metabolic data clusters in the NC and MCD groups were separated from each other; the similarity of the sample in each group was high; and the repeatability was good (Fig. 5B and C). These results indicated that the MCD diet significantly altered the metabolic profile of the intestinal flora. The metabolites identified by HMDB mainly included amino acids, carbohydrates, carbohydrate conjugates, fatty acids and conjugates, bile acids, alcohols, carbonyl compounds, hydroxycinnamic acids and derivatives, medium-chain hydroxy acids and derivatives, glycerophosphocholines, and fatty acyl glycosides; many of them were produced or regulated by the intestinal bacteria (Fig. 5D). KEGG functional prediction analysis of intestinal metabolites revealed that they were mainly involved in metabolic pathways including amino acid and lipid metabolism (Fig. 5E).

**Fig 6:**
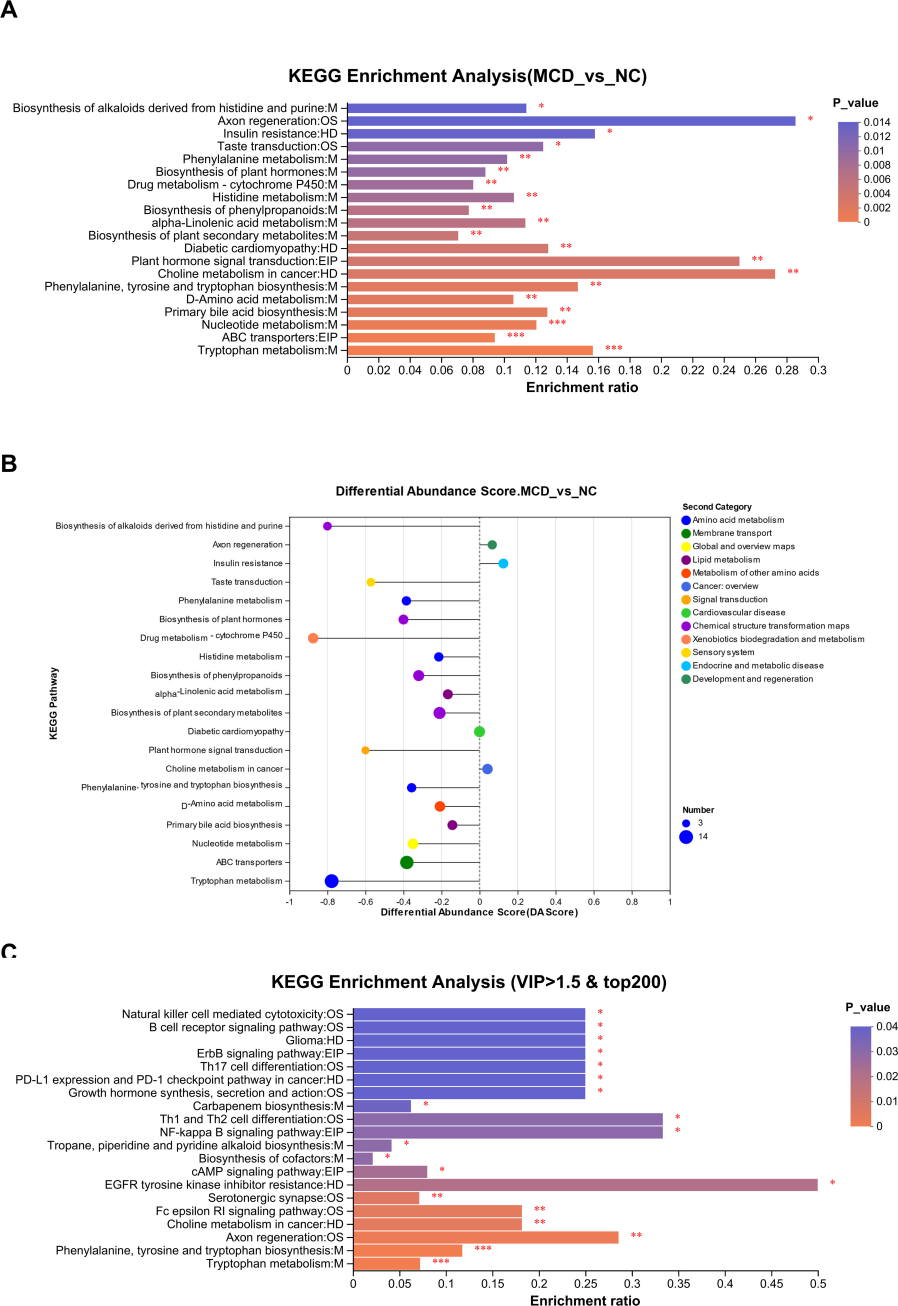
KEGG pathway enrichment analysis of differential metabolites. (**A**) KEGG pathway analysis of the 667 differential metabolites between the normal control (NC) and NAFLD (MCD) groups. The enrichment ratio represents the ratio of metabolite number enriched in this pathway to the background number annotated in the pathway. The higher the ratio, the greater the degree of enrichment. The column color gradient indicates the significance of the enrichment. The darker the default color, the more significantly enriched the KEGG term. *P (or false discovery rate [FDR]) <0.05, **P (or FDR) <0.01, ***P (or FDR) <0.001. (**B**) Differential abundance (DA) scores of the KEGG pathways. The DA score reflects the overall change of all metabolites in the metabolic pathway. A score of 1 indicates that the expression trend of all annotated differential metabolites in the pathway is upregulated; a score of –1 indicates that the expression trend of all annotated differential metabolites in the pathway is downregulated; the length of the line indicates the absolute value of the DA score. The size of the dot indicates the number of differential metabolites annotated in the pathway. The larger the dot, the more differential metabolites in the pathway. Dot distribution on the right side of the central axis and the longer line segment indicate that the overall expression of pathway trends is upregulated. The dots are distributed to the left of the central axis. The longer the line segment, the more inclined the overall downregulated expression of the pathway. (**C**) KEGG pathway analysis of the differential metabolites with top 200 of variable importance in projection of >1.5.

Figure 6A shows the different pathways of metabolite enrichment. The top 20 enriched KEGG pathways using BH multiple check correction showed that the pathways with differential metabolite enrichment in NAFLD mainly involved tryptophan metabolism, ABC transporters, nucleotide metabolism, primary bile acid biosynthesis, D-amino acid metabolism, phenylalanine, tyrosine and tryptophan biosynthesis, choline metabolism, diabetic cardiomyopathy, alpha-linolenic acid metabolism, biosynthesis of phenylpropanoids, histidine metabolism, drug metabolism - cytochrome P450, phenylalanine metabolism, taste transduction, insulin resistance, axon regeneration, and biosynthesis of alkaloids derived from histidine and purine. The differential abundance (DA) scores of the KEGG pathways were further analyzed (Fig. 6B), which revealed that all 14 metabolites were enriched in the tryptophan pathway and were more downregulated in MCD-fed mice than in NC mice. In addition to tryptophan metabolism, the other amino acid metabolism including histidine metabolism; phenylalanine metabolism; phenylalanine, tyrosine, and tryptophan biosynthesis pathway; and D-amino acid metabolism had a DA score of <0, indicating that the overall expression of the amino acid metabolism pathway was downregulated in the MCD group. Lipid metabolism, including primary bile acid biosynthesis and alpha-linolenic acid metabolism, was involved in MCD-induced development of NAFLD. Especially, the metabolites jasmonic acid, 9-oxo-non-anoic acid, traumatic acid, and dodecanedioic acid in the alpha-linolenic acid metabolism pathway were downregulated and traumatin and 7-epijasmonic acid were upregulated in the MCD group. It is worth mentioning that the compounds enriched in the insulin resistance pathway that were mainly upregulated were diradylglycerols and uridine diphosphate-N-acetylglucosamine in the MCD group, indicating that MCD-induced NAFLD in mice led to easier glucose absorption by regulating PDK1/GLUT4 signal transduction. Next, the top 200 VIP scores of >1.5 metabolites were evaluated to assess the signature, and KEGG pathway analysis was performed (Fig. 6C). The most enriched pathways were those involving the immune system, including the chemokine signaling pathway, Th1 and Th2 cell differentiation, Th17 cell differentiation, B-cell receptor signaling pathway, T-cell receptor signaling pathway, and natural killer cell-mediated cytotoxicity, which might be related to the occurrence of hepatitis induced by the long-term MCD diet. It might also be related to acidophilic necrosis and focal diffuse cell death as observed in the histopathological sections. The metabolites with KEGG pathway annotation information were selected for the subsequent analysis.

**Fig 7:**
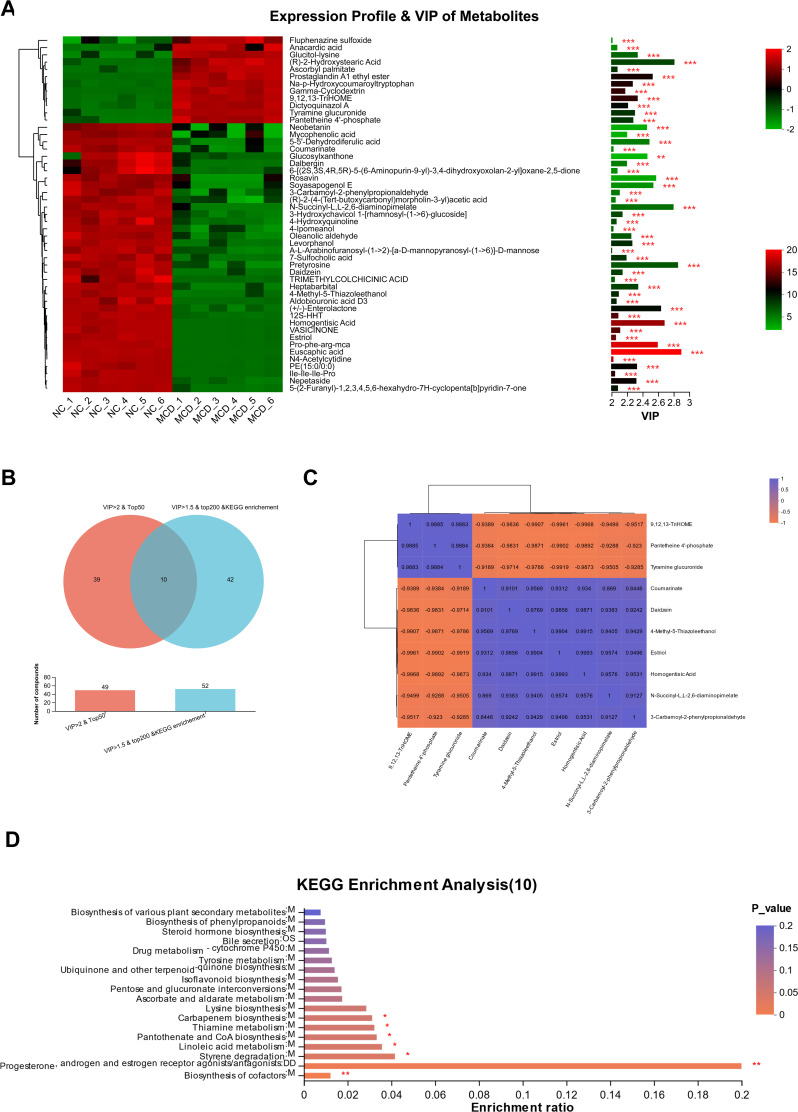
Significantly changed metabolites and markers in the NAFLD (MCD) group. (**A**) Top 50 metabolites with variable importance in projection (VIP) score of >2.0. (**B**) Venn diagram of the common metabolites of VIP score of >2 and top 50 with those of VIP >1.5 and top 200 and Kyoto Encyclopedia of Genes and Genomes (KEGG) annotation. (**C**) Correlation analysis of the 10 common metabolites using Pearson’s correlation coefficient by Euclidean distance algorithm. (**D**) KEGG analysis of these 10 common metabolites.The symbols ** and *** indicate that the *P*-value is less than 0.01 and 0.001, respectively.

The VIP score based on the PLS-DA model represented the potential of the metabolite as a biomarker, and the top 50 variables with a VIP score of >2.0 were considered important for the classification model (Fig. 7A). The metabolites with VIP scores of >2.0 and the top 50 variables were intersected with those with VIP scores of >1.5, and the top 200 and KEGG components that could be enriched into the KEGG functional pathway were analyzed and a total of 10 metabolites were obtained (Fig. 7B). These 10 metabolites shown in Fig. 7C reveal a strong positive or negative correlation among each other and mainly played roles in the pathway of biosynthesis of cofactors; progesterone, androgen, and estrogen receptor agonists/antagonists; styrene degradation; linoleic acid metabolism; pantothenate; CoA biosynthesis; thiamine metabolism; and carbapenem biosynthesis (Fig. 7D). These compounds, including 9,12,13-TriHOME in the linoleic acid metabolism pathway, might be potential biomarkers of liver injury in NAFLD or steatohepatitis.

### Correlation analysis between gut microbiota and metabolomic signatures

**Fig 8:**
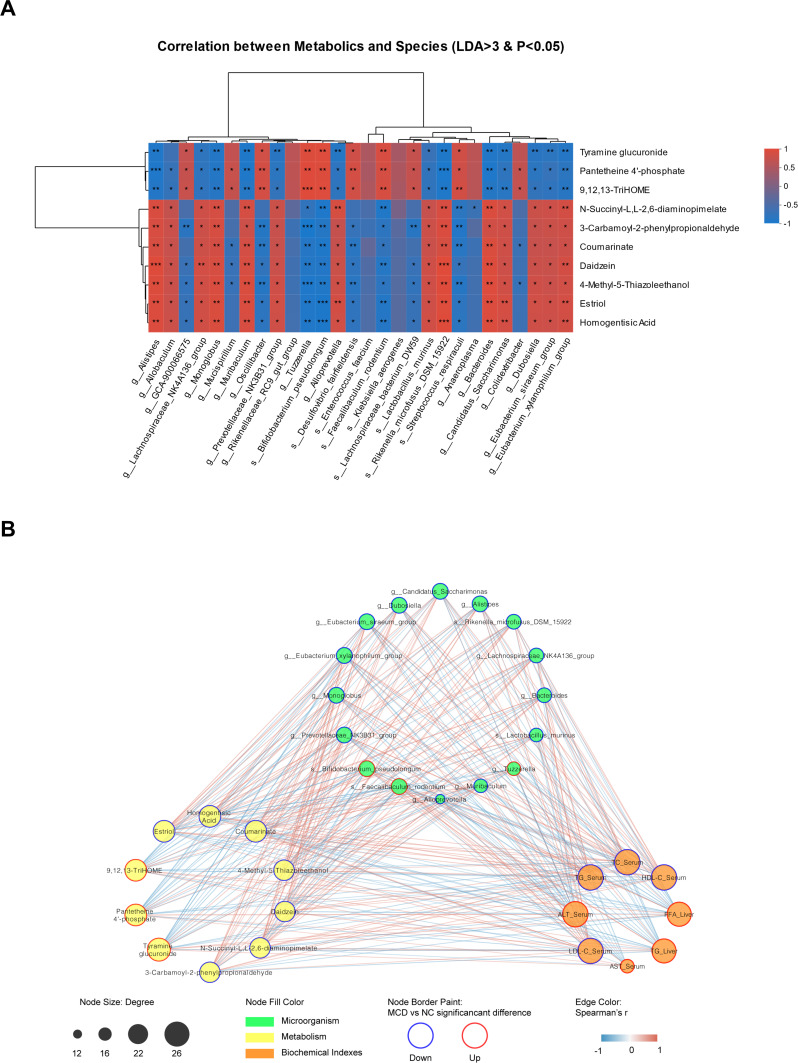
Correlation analysis between gut microbiota and metabolomic signatures. (**A**) Heatmap on the correlation among significantly altered gut microbiota and the differential metabolites using Pearson correlation analysis. Each cell in the figure represents the correlation between two attributes (metabolites and associated features), and different colors represent the size of the correlation coefficient between attributes. (**B**) Correlation of gut microbiota, metabolomic signatures, and biochemical indices using Cytoscape. Red represents upregulated metabolites and biochemical indices and the specific or predominant microbiota in the feces of MCD-fed mice, whereas blue represents the downregulated metabolites and biochemical indices or undetectable microbiota in the feces of MCD-fed mice.

A heatmap representing the correlations among significantly altered gut microbiota and the differential metabolites was plotted to identify the key gut bacteria and metabolites that potentially contribute to the development of non-obese NAFLD (Fig. 8A). Notably, except the species g_*Allobaculum*, g_*GCA-900066575*, g_*Mucispirillum*, g_*Oscillibacter*, g_*Rikenellaceae_RC9_gut_group*, s_*Desulfovibrio_fairfieldensis*, s_*Enterococcus_faecium*, s_*Klebsiella_aerogenes*, s_*Lachnospiraceae_bacterium_DW59*, g_*Anaeroplasma*, and g_*Colidextribacter*, the relative abundance of the other species with LDA of >3 and *P* < 0.05 showed a definite negative or positive correlation with the 10 metabolites that were obtained. It also indicated that the relative abundances of g_*Alistipes*, g_*Alloprevotella*, g_*Bacteroides*, g_*Candidatus_Saccharimonas*, g_*Dubosiella*, g_*Eubacterium_siraeum_group*, g_*Eubacterium_xylanophilum_group*, g_*Lachnospiraceae_NK4A136_group*, g_*Monoglobus*, g_*Muribaculum*, g_*Prevotellaceae_NK3B31_group*, s_*Lactobacillus_murinus*, and s_*Rikenella_microfusus_DSM_15922* were negatively correlated with tyramine glucuronide, pantetheine 4′-phosphate, and 9,12,13-TriHOME, and were positively correlated with coumarinate, 3-carbamoyl-2-phenylpropionaldehyde, N-succinyl-L,L-2,6-diaminopimelate, daidzein, 4-methyl-5-thiazoleethanol, homogentisic acid, and estriol, whereas the correlation between the relative abundance of g_*Tuzzerella*, s_*Bifidobacterium_pseudolongum*, s_*Faecalibaculum_rodentium*, and s_*Streptococcus_respiraculi* and these 10 metabolites was reversed. A table was also constructed, which only retained the significantly changed factors after manual inspection by further removing non-human relative metabolite components, including the plant metabolites coumarinate and daidzein (Fig. 8B). The red factors were upregulated in or were specific for MCD-fed mice, whereas the blue ones were downregulated or undetectable, collectively showing the 26 biomarkers characterizing the intestinal flora and intestinal metabolites of non-obese NAFLD.

## DISCUSSION

A comprehensive analysis of MCD-induced lean NAFLD was performed to evaluate the intestinal flora and intestinal metabolites. An MCD diet is widely used primarily because it reduces methyl donors and induces the full spectrum of NAFLD, which ranges from steatosis to liver fibrosis. Despite that, detailed reports on this model, which are often used as an auxiliary approach for verifications, are not available. Therefore, a more in-depth study of this animal model, as undertaken in this study, might potentially improve the understanding of the pathological mechanism regulating non-obese or lean NAFLD and contribute to the development of new drugs. Non-obese or lean-NAFLD disease and its models have unique characteristics that should not be ignored in contrast to the obese NAFLD model that has received considerable attention.

The levels of TG, FFA, and total fat in the liver were increased in lean mice with NAFLD, which were similar to the values reported for obese individuals with NAFLD (17). However, the lean-NAFLD mice showed decreased levels of serum TG, TC, HDL, LDL, and FFA (Fig. 2A through E). Although a significant difference in ALT and AST levels was found between the NC and MCD-fed groups, the levels of both were within the normal range (18), indicating that routine physical serum tests are unlikely to detect non-obese or lean NAFLD. Furthermore, NAFLD induced by the MCD diet causes lipid disorders, and drug treatment may result in several patterns of lipid homeostasis balance in the body. Although the effects of drug therapy on liver lipid content in MCD-induced NAFLD were evaluated in this study (19–21), the changes in serum lipid content have been rarely reported (22), probably because these changes are difficult to identify. Indeed, the same problem was encountered when this model was used for drug screening in our project (data not shown). Therefore, we hope that a further understanding of the model might aid in a more comprehensive pharmacodynamic analysis.

MCD-induced inflammation in mice with NAFLD has been reported in some detail (23). Our findings revealed that the levels of the biomarkers of inflammation such as TNF-α, IL-1β, IL-6, and IL-10 (Fig. 1F) were consistent with those reported previously. However, a comprehensive presentation of the gut microbiota and its metabolites is not yet available. Thus, our first focus was the upregulation of specific metabolite markers (tyramine glucuronide; 9,12,13-TriHOME, and pantetheine 4′-phosphate) in MCD-fed mice. Tyramine glucuronide is a naturally occurring metabolite of tyramine generated in the liver by UDP glucanosyltransferase and is involved in glucuronidation, which is toxic to hepatocytes. An increase in tyramine glucuronide has been reported in rats subjected to chronic ethanol treatment (24). 9,12,13-TriHOME is a trihydroxyoctadecenoic acid metabolite of linoleic acid, one of the major fatty acids found in lipids. TriHOMEs are linoleic acid-derived lipid mediators; 9,10,13-TriHOME is upregulated in high-fat diet (HFD)-induced NAFLD and indicates dysfunction in lipid metabolism (25). We found that 9,12,13-TriHOME was associated with MCD-induced NAFLD. Our findings are supported by the correlation of 9,12,13-TriHOME and high cholesterol in hypercholesterolemic rabbits (26). Pantetheine 4′-phosphate provides pantothenic acid for the acyl-carrier protein in the fatty acid synthase complex (27). High levels of pantetheine 4′-phosphate may lead to lipid metabolic disorder characterized by an increase in the synthesis of fatty acid synthase, which may promote NAFLD development or NASH progression (28). The relative expressions of the five metabolites, 3-carbamoyl-2-phenylpropionaldehyde, N-succinyl-L,L-2,6-diaminopimelate, 4-methyl-5-thiazoleethanol, homogentisic acid, and estriol, were markedly downregulated in MCD-fed mouse stools compared with those of NC mice. 3-Carbamoyl-2-phenylpropionaldehyde, N-succinyl-L,L-2,6-diaminopimelate, and 4-methyl-5-thiazoleethanol are the metabolites found in or produced by *Escherichia coli* (Pubchem website). They are related to metabolites of hepatorenal toxicity resulting from high doses of certain traditional Chinese medicines (29, 30). Homogentisic acid is a dihydroxyphenylacetic acid having hydroxy substituents at the 2- and 5-positions. It plays a role as a human and plant metabolite (Pubchem website), and it is also known as melanic acid. The accumulation of homogentisic acid in patients with alkaptonuria is associated with the concomitant deposition of pyomelanin (31). Our experiments also revealed a decrease in estriol in male mice with NAFLD. Some reports (32–34) show that increased estrogen levels lead to fatty liver by inhibiting fatty acid oxidation in the liver, promoting fatty acid synthesis and damaging mitochondria. Estradiol levels in adult male patients with NAFLD were found to be significantly higher than those of individuals in the control group and significantly positively correlated with homeostatic model assessment for insulin resistance, suggesting that increased estrogen levels may also lead to fatty liver by promoting insulin resistance. However, on the other hand, low estrogen levels can promote fatty liver disease, as evidenced by aromatase gene–knockout mice being unable to synthesize endogenous estrogen, resulting in lipid deposition in the liver (35). Estrogen also plays a key role in the maintenance of sex-specific expression of liver lipid β-oxidase series genome and the maintenance of liver lipid homeostasis (36). Thus, the expression of the eight metabolites (Fig. 8B) might indicate the specific characteristics of fecal metabolites in non-obese mice. These compounds associated with metabolic pathways, especially linoleic acid metabolism, might play a key role in the development of non-obese NAFLD.

Gut microbiota is the key to the treatment of NAFLD and other metabolic syndromes (37). The expression profiles of intestinal flora at the genus level were obtained based on relative abundance and LEfSe analysis with a relative abundance exceeding 1%. The MCD diet reduced microbial diversity, but individual species including g_Tuzzerella, s_*Bifidobacterium_pseudolongum*, s_*Faecalibaculum_rodentium*, and s*_Streptococcus_respiraculi* were still present at a significantly high relative abundance in the MFD diet-induced model of non-obese NAFLD. Our results were consistent with those of the article reporting that g_*Tuzzerella* was also predominant in the HFD-induced NAFLD group (38), suggesting it to be the key regulator in NAFLD progression. *Bifidobacterium pseudolongum* brings some benefits to individuals with obesity, such as TG reduction, by modulating gut microbiota in HFD-fed mice (39). In our study, the high abundance of *Bifidobacterium pseudolongum* in non-obese NAFLD might indicate its fat-reducing effect in the serum, which might be critical information for distinguishing between obese and non-obese NAFLDs. The abundance of s_*Faecalibaculum_rodentium* is increased in obese NAFLD and closely and positively correlated with hepatic lipids and immune cells (40). This might also be a characteristic of non-obese NAFLD, more specifically related to hepatic fat deposition. Our results were consistent with those reported previously. In addition, the relative abundance of the other 13 species (Fig. 8B) was extremely low, and some of them were almost undetectable in the MCD group. They are also extremely important for the diagnosis of non-obese NAFLD: the genus *Alistipes* is significantly increased in healthy subjects than in individuals with NAFLD (41); *Alloprevotella* is significantly decreased in the NAFLD treatment group (42); *Bacteroides* is more prevalent in gut dysbiosis and severe NAFLD (43); *Candidatus saccharimonas* is lower in mice with NAFL/NASH than in NC mice (42); the increase in the abundance of *Dubosiella* improves liver health by positively modulating the metabolite levels of dimethyl fumarate, lactitol, cafestol, and 4-hydroxyphenylacetic acid (44); *Eubacterium siraeum* abundance is reduced in patients with insulin resistance and is linked to NAFLD, whereas in non-human primates, its abundance is positively correlated with HDL-c levels (45, 46); *Eubacterium xylanophilum* is a potent butyrate-producing bacterium in the gut that is negatively associated with body weight and serum TC in HFD-fed mice (47); *Lachnospiraceae* NK4A136 group is higher in HFD-fed mice than in NC mice and comprises harmful bacteria (48); the abundance of the *Monoglobus* genera is relatively high in the normal diet group but low in the alcohol-induced mouse model of liver injury and in diabetic patients with a diagnosis of NAFLD (49, 50); *Muribaculum* is negatively correlated with short-chain fatty acids, which are important for intestinal integrity and are beneficial to the host, owing to their anti-obesity and anti-diabetic effects (51); the Prevotellaceae NK3B31 group is much more abundant in high (>5%)-liver fat groups than in low (<5%)-liver fat groups and serves as a gut microbiota biomarker of fatty liver disease (52); s_*Lactobacillus murinus* produces anti-bacterial compounds and exerts positive probiotic effects on host animals (53). Its abundance is reduced in patients with NAFLD having a body mass index ≥25 kg/m^2^ (54); s__*Rikenella_microfusus*_DSM_15922 is downregulated in diabetic animals and is regulated by *Rosa roxburghii* fruit polyphenol extracts; this species is a positive regulator of hepatic lipid synthesis and gluconeogenesis (55). The above analyses reveal that the changed flora identified in this study was correlated with NAFLD or fatty liver diseases. These bacteria are characteristic and typical of non-obese NAFLD. Among them, s_*Bifidobacterium_pseudolongum*, g_*Bacteroides*, g_*Lachnospiraceae_NK4A136_group*, and g_*Prevotellaceae_NK3B31_group* might be useful in further distinguishing between non-obese and lean-NAFLD microbiota.

### Conclusions

Fecal metabolomics was used for the identification of the key microbiota and the derived metabolites in non-obese NAFLD using MCD-induced male C57BL/6 J mice to establish a model of NAFLD. The high levels of tyramine glucuronide, 9,12,13-TriHOME, and pantetheine 4′-phosphate and the low levels of 3-carbamoyl-2-phenylpropionaldehyde, N-succinyl-L,L-2,6-diaminopimelate, 4-methyl-5-thiazoleethanol, homogentisic acid, and estriol might be useful biomarkers in the diagnosis of non-obese NAFLD. Among them, tyramine glucuronide, 9,12,13-TriHOME, and pantetheine 4′-phosphate, together with the predominant flora, including g_*Tuzzerella*, s_*Bifidobacterium pseudolongum*, and s_*Faecalibaculum rodentium*, were specific for non-obese mice with NAFLD and might be used as targets to identify drugs to treat non-obese NAFLD. From a holistic viewpoint, g_*Alistipes*, g_*Alloprevotella*, g_*Bacteroides*, g_*Candidatus_Saccharimonas*, g_*Dubosiella*, g_*Eubacterium_siraeum_group*, g_*Eubacterium_xylanophilum_group*, g_*Lachnospiraceae_NK4A136_group*, g_*Monoglobus*, g_*Muribaculum*, g_*Prevotellaceae_NK3B31_group*, s_*Lactobacillus_murinus*, and s_*Rikenella_microfusus_DSM_15922* might be important and distinctive components in non-obese NAFLD versus those in normal and obese fatty liver. Gut microbiota and the derived metabolites are central regulators of metabolic disorders including NAFLD. Thus, the analysis of gut microbiota and their metabolites might be an effective methodology to screen therapeutic agents and biomarkers for the prognosis and diagnosis of NAFLD, supporting human-associated results found in subjects with non-obese NAFLD. It is, therefore, particularly significant for individuals with non-obese NAFLD who need to be more actively monitored.

## Data Availability

The data are available from the authors upon reasonable request. Raw sequence data of the microbiota have been deposited into National Center for Biotechnology Information’s Sequence Read Archive under accession number PRJNA1064694. Raw liquid chromatography-tandem mass spectrometry data of the fecal metabolites have been made available on the MetaboLights database, under accession number MTBLS9365.
